# Radiosynthesis and Preclinical Evaluation of [^18^F]F-DPA, A Novel Pyrazolo[1,5a]pyrimidine Acetamide TSPO Radioligand, in Healthy Sprague Dawley Rats

**DOI:** 10.1007/s11307-016-1040-z

**Published:** 2017-01-12

**Authors:** Thomas Keller, Anna Krzyczmonik, Sarita Forsback, Francisco R. López Picón, Anna K. Kirjavainen, Jatta Takkinen, Johan Rajander, Fanny Cacheux, Annelaure Damont, Frédéric Dollé, Juha O. Rinne, Merja Haaparanta-Solin, Olof Solin

**Affiliations:** 1 Radiopharmaceutical Chemistry Laboratory, Turku PET Centre, University of Turku, Turku, Finland; 2 Department of Chemistry, University of Turku, Turku, Finland; 3 PET Preclinical Imaging Laboratory, Turku PET Centre, University of Turku, Turku, Finland; 4 MediCity Research Laboratory, University of Turku, Turku, Finland; 5 Accelerator Laboratory, Turku PET Centre, Åbo Akademi University, Turku, Finland; 6 CEA, I2BM, Service Hospitalier Frédéric Joliot, Orsay, France; 7 Turku PET Centre, Division of Clinical Neurosciences, Turku University Hospital, Turku, Finland; 8 Turku PET Centre, University of Turku, Kiinamyllynkatu 4-8, 20520 Turku, Finland

**Keywords:** TSPO, Electrophilic fluorination, F-DPA, DPA-714, Pyrazolopyrimidine, Fluorine-18, Neuroinflammation, Brain, PET, [^18^F]Selectfluor bis(triflate)

## Abstract

**Purpose:**

Many neurological conditions result in the overexpression of the translocator protein 18 kDa (TSPO), today recognized as a biomarker for microglial activation and neuroinflammation imaging. The pyrazolo[1,5-a]pyrimidine acetamides are a particularly attractive class of TSPO-specific ligands, prompting the development of several positron emission tomography (PET) radiotracers. This includes F-DPA, a recently reported fluorinated ligand (*K*_i_ = 1.7 nM), wherein the fluorine atom is directly linked to the phenyl moiety without the presence of an alkyl or alkoxy spacer chain. Reported here is the preparation of [^18^F]F-DPA using [^18^F]Selectfluor bis(triflate) and the preliminary evaluation of [^18^F]F-DPA in healthy rats. Its metabolic profile and biodistribution in rats are compared with that of [^18^F]DPA-714, a closely related structure.

**Procedures:**

[^18^F]F-DPA was synthesized by electrophilic fluorination using [^18^F]Selectfluor bis(triflate), [^18^F]DPA-714 was synthesized by conventional nucleophilic fluorination. The biodistribution of both radiotracers was compared in Sprague Dawley rats. Radiometabolites of both radiotracers in plasma and brain homogenates were analyzed by radioTLC.

**Results:**

The radiochemical yield of [^18^F]F-DPA was 15 ± 3 % and the specific activity was 7.8 ± 0.4 GBq/μmol. The radiochemical purity exceeded 99 %. The *in vivo* time activity curves of [^18^F]F-DPA demonstrate rapid entry into the brain and a concentration equilibrium at 20–30 min after injection. The metabolic profiles at 90 min after radiotracer injection in the plasma show that unchanged [^18^F]F-DPA and [^18^F]DPA-714 account for 28.3 ± 6.4 and 11.1 ± 2.6 % of the remaining radioactivity, respectively. In the brain, unchanged [^18^F]F-DPA accounts for 93.5 ± 2.8 % of the radioactivity; whereas for [^18^F]DPA-714, this value is 53.6 ± 1.6 %.

**Conclusions:**

[^18^F]Selectfluor bis(triflate) was successfully used to label F-DPA with fluorine-18. The labeling position on the aromatic moiety imparts a higher stability compared to [^18^F]DPA-714 with regard to *in vivo* metabolism. [^18^F]F-DPA is a promising new radiotracer and warrants further investigation in animal models of disease.

**Electronic supplementary material:**

The online version of this article (doi:10.1007/s11307-016-1040-z) contains supplementary material, which is available to authorized users.

## Introduction

The translocator protein 18 kDa (TSPO), localized on the outer mitochondrial membrane [1], plays a crucial role in the transport of cholesterol across the aqueous intermembrane space into the mitochondrial matrix [2]. Within the mitochondrion, cholesterol is used in the synthesis of steroids and creation of new mitochondrial membrane during cell multiplication and repair.

In recent years, TSPO has gained much attention as a biomarker for neuroinflammation and microglial activation due to its overexpression within the brain of individuals suffering from neurological conditions such as Alzheimer’s disease, multiple sclerosis, stroke, and traumatic brain injury [3]. In addition, the location of TSPO on the outer mitochondrial membrane makes it an attractive and readily accessible target for low molecular weight molecules. As a result, numerous TSPO-specific ligands have been developed and radiolabeled for use in positron emission tomography (PET) [4] and single photon emission computed tomography [5]. One of the most significant ligands for TSPO is the isoquinolinecarboxamide PK11195 (*N*-butan-2-yl-1-(2-chlorophenyl)-*N*-methylisoquinoline-3-carboxamide). It was first labeled with tritium in 1985 by Inoue *et al*. [6] and later with carbon-11 by Camsonne *et al*. [7]. Despite the high lipophilicity and slow wash out of PK11195 compared to the second generation TSPO ligands, [^11^C]PK11195 has not been phased out from the clinical setting and still remains a standard which is routinely used in the evaluation of novel TSPO-specific PET ligands.

Since the discovery of PK11195, a range of motifs have been identified as alternative TSPO ligands and labeled for PET imaging. These include the atypical benzodiazepine [^11^C]Ro5-4864 [8], the vinca alkaloid [^11^C]Vinpocetine [9], dihydro-9H-purinacetamides (for example, *N*-benzyl-*N*-ethyl-2-(7-[^11^C]methyl-8-oxo-2-phenylpurin-9-yl)acetamide, known as [^11^C]Emapunil [10]), aryloxyanilides (such as [^11^C]PBR28 [11] and [^18^F]FEPPA [12]), indoleacetamides (such as [^18^F]GE-180 [13] and [^11^C]SSR180575 [14]), acetamidobenzoxazolones [15] without forgetting pyrazolopyrimidines, and bioisoteric structures (imidazopyridines, for example). The latter classes indeed include an impressive list of PET-radioligands, among them, the 5,7-dimethylpyrazolo[1,5-a]pyrimidin-3-yl)acetamides [^11^C]DPA-713 [16], [^18^F]DPA-714 [17], and [^18^F]PBR146 [18], and the 6-chloro-imidazo[1,2-a]pyridin-3-yl)acetamides [^11^C]CLINME [19], [^18^F]PBR102 [18] and [^18^F]PBR111 [20].

The use of the short-lived carbon-11 label (*t*_1/2_ = 20.4 min) in the cases of [^11^C]CLINME and [^11^C]DPA-713 limits their use to facilities with an on-site cyclotron. While the fluorine-18-labeled tracers, [^18^F]PBR102, [^18^F]PBR111, [^18^F]PBR146, and [^18^F]DPA-714, have good brain penetration and kinetics, their main pitfall arises from their labeling positions. Metabolism of these tracers results in cleavage of the radioactive label [21, 22] and the formation of non-specifically binding radiometabolites. Despite these shortcomings, the extensive use of these tracers in both preclinical and clinical studies, together with the ongoing development of more metabolically stable analogues [23, 24], further underpins the importance of this motif.

The short half-life of C-11-labeled radiotracer can be both advantageous as well as a drawback. Carbon-11-labeled analogues of bioactive molecule can be produced without modifying their structure. Additionally, the short half-life of carbon-11 makes it possible to image a test subject multiple times in one day. However, the short half-life also limits the clinical applicability of these tracers due to the time required for distribution to PET centers that do not have an on-site cyclotron. Fluorine-18 chemistry for PET imaging has attracted interest since the half-life of this radioisotope (*t*_1/2_ = 109.8 min) is long enough to allow late-stage reactions and purification to be performed. Additionally, a single production batch can be used for multiple patients provided that the radiotracer is stable with respect to radiolysis. Finally, it is possible to transport fluorine-18-labeled radiotracers to PET facilities that do not have an on-site cyclotron. At the same time the radioisotope decays quickly enough, like all short-lived emitters, that the body-concentration of the radioisotope falls to zero in a matter of hours. Due to the low energy (*E*_βmax_ = 0.634 MeV) of the positrons emitted, imaging using fluorine-18-labeled radiotracers gives high resolution PET images, which is of particular importance when imaging the brain and especially at the preclinical stage in studies using small animals.

**Fig. 1:**
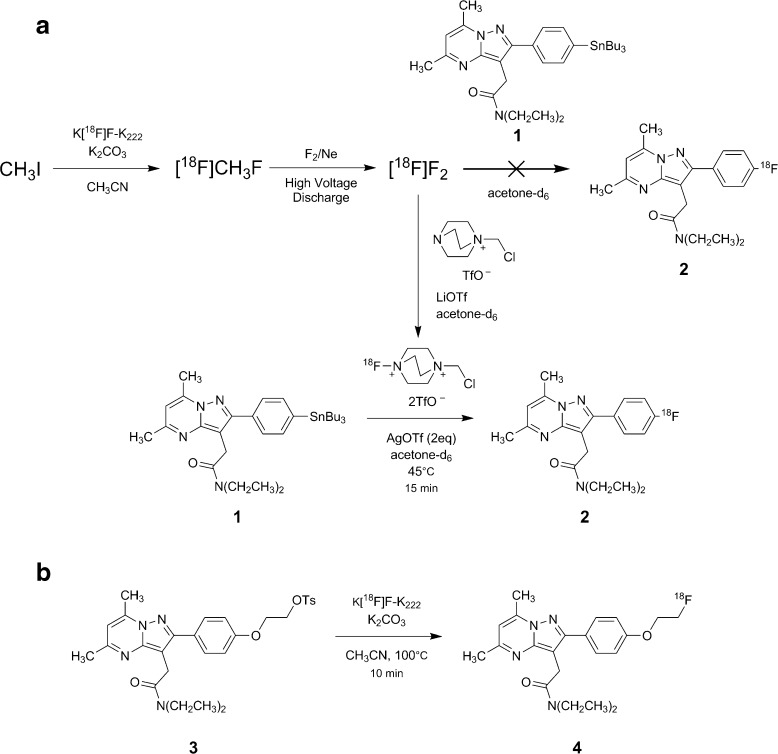
Scheme showing radiochemical syntheses used for the preparation of both radiotracers. **a** Production of [^18^F]F_2_, the attempted direct labeling reaction using the reagent, production of [^18^F]Selectfluor bis(triflate), and synthesis of [^18^F]F-DPA using electrophilic fluorination. **b** Nucleophilic fluorination of [^18^F]DPA-714 using the conventional, no-carrier-added Kryptofix 222/K^+^[^18^F]F^−^ complex.

*N,N*-Diethyl-2-(2-(4-fluorophenyl)-5,7-dimethylpyrazolo[1,5-a]pyrimidin-3-yl)acetamide (F-DPA, Fig. 1a, compound **2**) is a fluorine-containing pyrazolopyrimidine with a structure closely-related to DPA-714, also showing a good affinity (*K*_i_ = 1.7 nM) and selectivity (*K*_i_ CBR > 1 μM) [25] towards the TSPO. In compound **2**, the alkoxy-chain bridging the phenyl group and the fluorine atom has been removed, thus positioning the fluorine directly on the phenyl moiety. As such, improved metabolic stability of the radiotracer would be expected, and notably, the formation of small fluorine-18-labeled radiometabolites that enter the brain, poorer PET imaging quality and restrained quantitative imaging, as demonstrated for [^18^F]DPA-714 [21], should be avoided.

Reported herein is the use of a mild electrophilic [^18^F]fluorination reagent, 1-(chloromethyl)-4-[^18^F]fluoro-1,4-diazoniabicyclo[2.2.2]octane bis(triflate) ([^18^F]Selectfluor bis(triflate)) [26] for the preparation of [^18^F]F-DPA, and the evaluation of this radiotracer in healthy rats. The metabolic profile and biodistribution of [^18^F]F-DPA were compared with that of the closely-related radiotracer [^18^F]DPA-714.

## Materials and Methods

Details of the chemicals and HPLC systems used during the radiochemical syntheses are provided in the Supplementary Material.

### F-DPA, DPA-714, and Related Precursors for Labeling

*N,N*-Diethyl-2-(2-(4-fluorophenyl)-5,7-dimethylpyrazolo[1,5-a]pyrimidin-3-yl)acetamide (F-DPA, Fig. 1a, compound **2**), *N,N*-diethyl-2-(2-(4-(2-fluoroethoxy)phenyl)-5,7-dimethyl-pyrazolo[1,5-α]pyrimidin-3-yl)acetamide (DPA-714, Fig. 1b, compound **4**), and *N,N*-diethyl-2-(2-(4-(2-tosylethoxy)phenyl)-5,7-dimethyl-pyrazolo[1,5-α]pyrimidin-3-yl)acetamide (DPA-714 labeling precursor, Fig. 1b, compound **3**) were synthesized as described by Damont *et al*. [25, 27]

*N,N*-Diethyl-2-(2-(4-(tributylstannyl)phenyl)-5,7-dimethyl-pyrazolo[1,5-α]pyrimidin-3-yl)acetamide (F-DPA labeling precursor, Fig. 1a, compound **1**) was synthesized from *N,N*-diethyl-2-(2-(4-iodophenyl)-5,7-dimethylpyrazolo[1,5-*a*]pyrimidin-3-yl)acetamide according to the procedure described in the Supplementary Material.

### Radionuclide Production

Fluorine-18 was produced as [^18^F]fluoride via the ^18^O(*p,n*)^18^F nuclear reaction on an ^18^O-18-enriched water target, a detailed description is provided in the Supplementary Material.

### Labeling Syntheses

#### [^18^F]F_2_ and [^18^F]Selectfluor bis(triflate) syntheses

[^18^F]F_2_ gas was synthesized according to a similar procedure as described by Bergman and Solin [28].

[^18^F]Selectfluor bis(triflate) was synthesized according to the method reported by Teare *et al*. [25]. These procedures are described in detail in the Supplementary Material.

### Direct Labeling with [^18^F]F_2_

In initial labeling tests, [^18^F]F_2_ was bubbled through a solution of *N,N*-diethyl-2-(2-(4-(tributylstannyl)phenyl)-5,7-dimethyl-pyrazolo[1,5-α]pyrimidin-3-yl)acetamide (F-DPA labeling precursor, 3.1 mg (4.9 μmol), Fig. 1a, compound **1**) in freon-11 (CCl_3_F, 700 μl) or acetone-*d*_6_ (750 μl) with AgOTf (3 mg, 12 μmol) as an additive.

### Labeling with [^18^F]Selectfluor bis(triflate)

Further labeling tests were performed using [^18^F]Selectfluor bis(triflate) synthesized from [^18^F]F_2_. For this, F-DPA labeling precursor (compound **1**, 2.6 ± 0.7 mg (4.1 ± 1.2 μmol)) and AgOTf (2 equivalents) were added to typically 200 μl of [^18^F]Selectfluor bis(triflate) in acetone-*d*_6_ (with a total radioactivity of 500 ± 13 MBq; Fig. 1a). The reaction mixture was stirred at 45 °C and samples for HPLC analysis were taken after 15 and 60 min of reaction.

Analytical HPLC was performed using a Merck Chromolith Performance RP-18e (10 μm, 4.6 × 100 mm) column (Merck KGaA, Darmstadt, Germany) with an isocratic eluent system consisting of 70 % 0.025 M aq. NaH_2_PO_4_ (pH 3.5) and 30 % CH_3_CN and a flow rate of 4 ml/min. Using these conditions, [^18^F]F-DPA showed a 5.5 min retention time.

The effect of a higher concentration of reactants was also investigated. For this, after the addition of the crude stock of [^18^F]Selectfluor bis(triflate) (200 μl), half of the acetone was evaporated under a helium flow. The reaction was also sampled at 15 and 30 min, and analyzed with HPLC using the conditions previously mentioned. In each case, the [^18^F]F-DPA fraction (retention time 5.5 min) was collected, the radioactivity of the fraction measured and the radiochemical yield was calculated.

The conditions chosen for the production of [^18^F]F-DPA batches for preclinical evaluation were as described above, with double concentration of reagents and a reaction time of 15 min. 4.0 ± 1.6 mg (6.4 ± 2.6 μmol) of F-DPA precursor for labeling were used, together with the entire crude stock (7.5 ± 0.8 GBq) of the [^18^F]Selectfluor bis(triflate) produced.

Preparative HPLC of the crude reaction mixture was also performed, and this using a Waters X-Terra Prep RP18 (10 μm, 7.8 × 300 mm) column (Waters Corporation, Milford MA, USA) and an isocratic eluent system consisting of 65 % 0.1 M aq. CH_3_CO_2_NH_4_ and 35 % CH_3_CN, and a flow rate of 4 ml/min. Using these conditions, [^18^F]F-DPA had a retention time of 22–23 min. The fraction containing the product was collected, diluted with water (25 ml), and passed over a Waters Sep-Pak Light tC18 cartridge (Waters Corporation, Milford, MA, USA), thereby trapping the radioligand. The cartridge was then washed with water (25 ml). [^18^F]F-DPA was finally eluted using ethanol (400 μl) and further diluted with saline to give a 10 % ethanolic solution, suitable for intravenous injection. Analysis of the [^18^F]F-DPA batches was carried out using the same previously described analytical HPLC system and conditions.

### Production of [^18^F]DPA-714

[^18^F]DPA-714 was synthesized according to a procedure similar to that previously described by James et al. [17]. The synthesis is described in detail in the Supplementary Material.

### Mass Spectroscopy

The crude product mixtures resulting from direct fluorination reactions were analyzed by liquid chromatography coupled to mass spectrometry (QTrap, Applied Biosystems MIDS SCIEX, Toronto, Canada). The HPLC system used included a Merck Chromolith Performance RP-18e (10 μm, 4.6 × 100 mm) column and an isocratic eluent system consisting of 0.1 % aq. formic acid and CH_3_CN (70:30), followed by a 50 % CH_3_CN wash. The flow rate was constant at 4 ml/min.

### Animals and Ethical Statement

The study was approved by the Regional State Administrative Agency for Southern Finland (license number ESAVI/3899/04.10.07/2013). Animal studies were performed on male Sprague Dawley rats (total *n* = 36, [^18^F]DPA-714; *n =* 18, [^18^F]F-DPA; *n* = 18, weight 290 ± 30 g).

All animals were group-housed under standard conditions (temperature 21 ± 3 °C, humidity 55 ± 15 %, lights on from 6:00 a.m. until 6:00 p.m.) at the Central Animal Laboratory, University of Turku, Turku, Finland.

### *In Vivo* PET/CT Imaging and Analysis of PET Data

Rats were anesthetized with isoflurane/oxygen gas and injected intravenously with [^18^F]F-DPA (31.4 ± 2.1 MBq, *n* = 4 SA at the time of injection = 4.1 ± 0.4 GBq/μmol) for scanning by an Inveon multimodality PET/CT tomograph (Siemens Medical Solutions, Knoxville, TN, USA). In order to prevent eye dryness, a few drops of Oftagel (25 mg/g; Santen, Tampere, Finland) were applied to the eyes of the animals. The scanner has a 12.7 cm axial field of view (FOV) and 10 cm transaxial FOV generating images from 159 transaxial slices. The spatial resolution of the scanner is 1.4 mm according to the manufacturer’s specifications. The animals were scanned for 10 min with computed tomography (CT) for attenuation correction and anatomical reference, and immediately after that, the radiotracer was injected for a 60-min dynamic PET scan. Frames were taken at the following intervals: 30 × 10, 15 × 60, 4 × 300, and 2 × 600 s. Volume of interests (VOIs) were drawn over the whole brain, heart, lung, liver, and kidneys using Inveon Research Workplace Image Analysis software (Siemens Medical Solutions). From the VOIs, time–activity curves (TACs) were obtained and the uptake of [^18^F]F-DPA was expressed as percentage of injected dose per milliliter of tissue (%inj. dose/ml).

### *Ex Vivo* Biodistribution of [^18^F]F-DPA and [^18^F]DPA-714

For biodistribution studies, the anesthetized animals were injected with [^18^F]F-DPA (30.7 ± 1.5 MBq) or [^18^F]DPA-714 (29.0 ± 2.1 MBq). After sacrifice (5, 15, 30, 60, and 90 min after injection, *n* = 3, for every time point and radiotracer), the blood was collected into Li-heparin tubes and transcardial perfusion with saline was conducted rapidly in order to eliminate the blood from the whole body. Following this, the organs of the animals were collected, individually weighed, and the radioactivity was measured using a gamma counter (Wizard^2^ 3 × 3″, PerkinElmer, Turku, Finland). The decay-corrected radioactivity is reported as percentage of injected dose per gram (%ID/g).

### Plasma Protein Binding

For the plasma protein binding studies, the anesthetized animals (*n* = 3 for each tracer) were injected with [^18^F]F-DPA (27.0 ± 5.1 MBq) or [^18^F]DPA-714 (29.8 ± 3.4 MBq). After 15 min, the animals were sacrificed and the blood was collected into Li-heparin tubes and centrifuged (3000×*g*, 10 min). A 10-μl aliquot of the plasma was spotted onto a TLC plate; following this, the remaining plasma was filtered by ultrafiltration using Microsep 30K Omega (Pall Life Sciences, Mexico) with a cut-off of 30 kDa. A 10-μl aliquot of the resulting supernatant was also spotted onto the TLC plate. The plates were exposed onto an imaging plate (Fuji BAS Imaging Plate TR2025, Fuji Photo Film Co., Ltd., Tokyo, Japan) for approximately two half-lives of fluorine-18 (3.5 to 4 h). After the exposure, the imaging plates were scanned using a BAS-5000 reader (Fuji, Japan) with a resolution of 50 μm and the saved images were analyzed using TINA software (version 2.10 g, Raytest, Isotopenmessgeräte, GmbH, Straubenhardt, Germany).

### RadioTLC Analyses of [^18^F]F-DPA and [^18^F]DPA-714 Radiometabolites

The blood samples were taken into Li-heparin tubes and centrifuged (3000×*g*, 10 min). The plasma was transferred to an Eppendorf tube and the plasma proteins were precipitated by addition of 1.5 parts of MeOH by volume. Following a further centrifugation (12,000×*g*, 4 min), 20 μl of the supernatant was spotted onto an aluminum-backed silica gel 60/Kieselguhr F_254_ TLC plate (EMD Millipore 1.05567.0001, Merck Millipore, Darmstadt, Germany).

Brain samples from the cortex were homogenized with approximately 200 μl of 1:1 *v*/*v* of MeOH and water; the mixture was transferred to an Eppendorf tube and centrifuged (12,000×*g*, 4 min). An aliquot (20 μl) of the supernatant was spotted onto the TLC plate.

The TLC plates were developed with a mixture of dichloromethane and methanol (9:1, *v*/*v*) for a distance of 6 cm. Following this, the plates were exposed onto an imaging plate (Fuji BAS Imaging Plate TR2025, Fuji Photo Film Co., Ltd., Tokyo, Japan) for approximately two half-lives of fluorine-18 (3.5 to 4 h). After the exposure, the imaging plates were scanned using a BAS-5000 reader (Fuji, Japan) with a resolution of 50 μm and the saved images were analyzed using TINA software (version 2.10 g, Raytest, Isotopenmessgeräte, GmbH, Straubenhardt, Germany).

### Statistical Methods

The results are reported as means ± SD (*n* ≥ 3) or as individual values (*n* < 3). All statistical analyses were performed using the Graph-Pad Prism program (version 6.04; GraphPad Software). Differences between the *ex vivo* blood activities (%ID/g) for the two radiotracers were tested using the two-tailed unpaired *t* test. Differences were considered statistically significant if the *P* value was less than 0.05.

## Results

### Radiochemistry

Labeling of [^18^F]F-DPA was initially attempted directly using the discharge-produced [^18^F]F_2_. However, when using either freon-11 or acetone-*d*_6_ (with silver triflate) as the solvent, [^18^F]F-DPA was not produced and only fluorine-18-labeled, but still stannylated by-products were obtained. Indeed, molecular ions with masses corresponding to mono, di, tri, and tetra-fluorination of F-DPA’s precursor were observed. However, the labeling positions in these products could not be identified by LC/MS. [^18^F]Selectfluor bis(triflate) was thus employed as an alternative [^18^F]fluorinating reagent, and optimized conditions for the synthesis of [^18^F]F-DPA were determined as shown in Table 1.

**Table 1:** Summary of the screening reactions

Entry	Reaction time (min)	Volume of reaction mixture (μl)	Radioactivity of [^18^F]Selectfluor bis(triflate) (GBq)	RCC (%)
1	15	200	0.518	33
2	60	200	0.518	27
3	15	100	0.495	31
4	30	100	0.495	44
5	15	100	0.497	37
6	30	100	0.497	31

*RCC* radiochemical conversion, radioactivity of product fraction collected from HPLC expressed as a percentage of the total injected activity (both activities are decay-corrected to the EOB)

For preclinical evaluation, a 15-min reaction time and a double concentration of reactants were the conditions selected. [^18^F]F-DPA was produced from 7.5 ± 0.8 GBq of [^18^F]Selectfluor bis(triflate) in 15 ± 3 % radiochemical yield (radioactivity of product, decay-corrected to end of bombardment (EOB), collected from HPLC, expressed as a percentage of the [^18^F]Selectfluor bis(triflate) radioactivity, also decay-corrected to EOB). SA (decay-corrected to EOB too) was 7.8 ± 0.5 GBq/μmol and the radiochemical purity exceeded 99 %.

[^18^F]DPA-714 was synthesized in 43 ± 7 % radiochemical yield, relative to starting [^18^F]fluoride activity, with SA greater than 1 TBq/μmol, and a radiochemical purity also exceeding 99 %.

### *In Vivo* PET/CT Imaging and Analysis of PET data

**Fig. 2:**
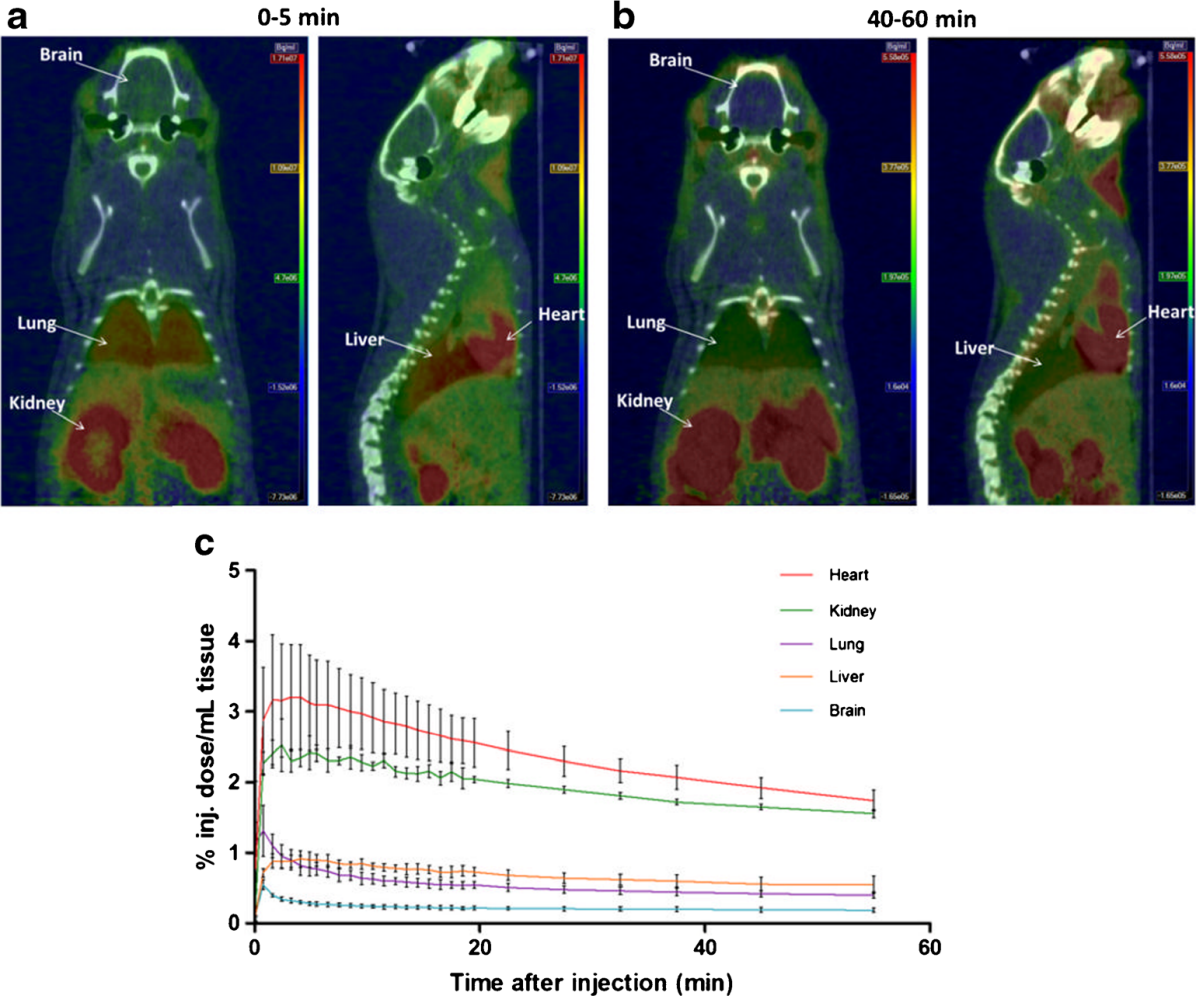
Representative PET/CT images from a Sprague Dawley rat are summed at **a** 0–5 and **b** 40–60 min after injection of [^18^F]F-DPA. **c** Time-activity curves of [^18^F]F-DPA obtained from VOIs in the whole brain, lungs, heart, liver, and kidneys. Mean ± SD, *n* = 3.

In the whole-body *in vivo* representative distribution of [^18^F]F-DPA in a Sprague Dawley (SD) rat (Fig. 2a, b), the mean TACs for [^18^F]F-DPA demonstrated that the radiotracer rapidly entered into the brain with an equilibrium concentration reached at approximately 20–30 min after injection (Fig. 2c). At the periphery, the *in vivo* TACs showed that [^18^F]F-DPA was excreted via the hepatobiliary pathway based on the high levels of F-18 radioactivity that accumulated in the intestine.

### *Ex Vivo* Biodistribution of [^18^F]F-DPA and [^18^F]DPA-714

**Fig. 3:**
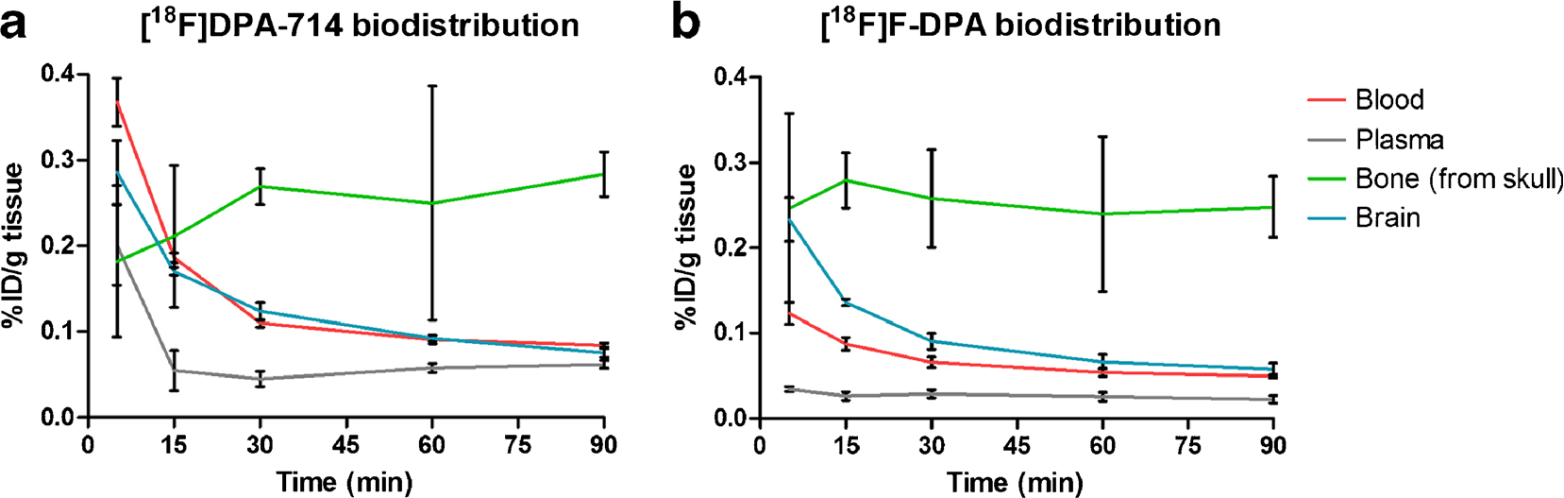
Measurement of the uptake of **a** [^18^F]F-DPA and **b** [^18^F]DPA-714 in the brain, blood, plasma, and bone derived from *ex vivo* biodistribution data performed on Sprague Dawley rats (mean ± SD, *n* = 3 per time point).

The full *ex vivo* biodistribution data for [^18^F]F-DPA and [^18^F]DPA-714 in SD rats at 5, 15, 30, 60, and 90 min after injection are given in Supplementary Table 1. Measurement of the uptake of (a) [^18^F]F-DPA and (b) [^18^F]DPA-714 in the brain, whole blood, plasma, and bone (Fig. 3) showed that after the initial uptake, measured at 5 min, there was no significant accumulation of radioactivity in the bone with either of the radiotracers, which suggests that [^18^F]F-DPA and [^18^F]DPA-714 are not rapidly defluorinated *in vivo*. A clear wash-out of radioactivity from the blood and brain was seen. In the case of [^18^F]F-DPA, the blood radioactivity was significantly lower (*P* ≤ 0.006) than that of [^18^F]DPA-714.

At the periphery, the highest levels of radioactivity for both radiotracers were measured in the adrenal glands, spleen, heart, lungs, kidneys, and small intestine (Suppl. Table 1).

### Plasma Protein Binding

The plasma binding study showed that at 15 min after injection of the tracers, the fraction of radioactivity unbound to the plasma proteins is 6.6 ± 3.1 % in the case of [^18^F]F-DPA while for [^18^F]DPA-714 the free radioactivity fraction is 32.8 ± 8.8 %.

### RadioTLC Analyses of [^18^F]F-DPA and [^18^F]DPA-714 Radiometabolites

**Fig. 4:**
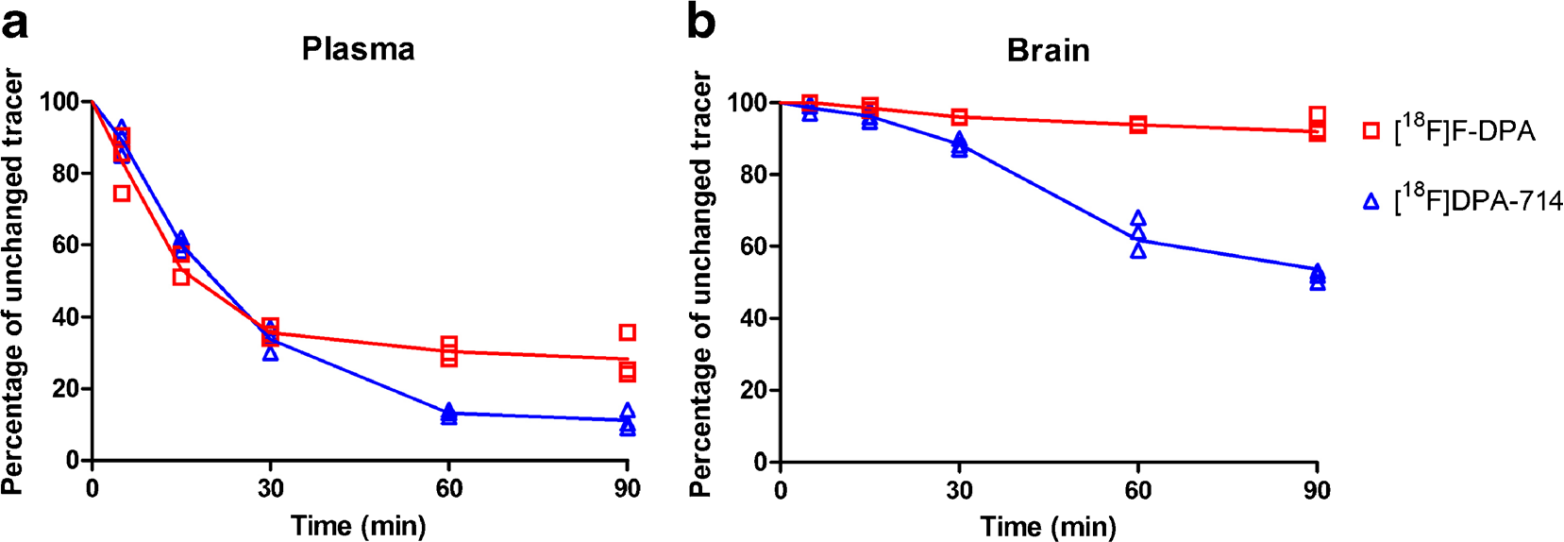
Comparison of the percentage of unchanged [^18^F]F-DPA and[^18^F]DPA-714 in Sprague Dawley rat **a** plasma and **b** brain, measured by radioTLC, at different time points after radiotracer injection (*n* = 3 per time point).

The radioTLC analyses of the plasma following radiotracer injection demonstrated that there was a degree of metabolism for both [^18^F]F-DPA and [^18^F]DPA-714 (Fig. 4). [^18^F]F-DPA, retention factor (*R*_f_) = 0.83, had two major radiometabolites that were more polar than the parent compound, with *R*_f_ values of 0.60 and 0.67 (Suppl. Fig. 1). In the case of [^18^F]DPA-714, there were also two major radiometabolites (Suppl. Fig. 2), one with a slightly lower *R*_f_ value (*R*_f_ = 0.67) compared to the unchanged tracer (*R*_f_ = 0.78) and a second that did not move from the origin (*R*_f_ = 0). At 90 min after injection of [^18^F]F-DPA, 28.3 ± 6.4 % of the radioactivity in the plasma could still be attributed to unmetabolized [^18^F]F-DPA, while in the case of [^18^F]DPA-714, at the same time point, the unchanged radiotracer accounted for only 11.1 ± 2.6 % of the remaining radioactivity (Fig. 4a). At 90 min after injection, 93.5 ± 2.8 % of the radioactivity in the brain was attributed to unmetabolized [^18^F]F-DPA, while for [^18^F]DPA-714, at the same time point, only 53.6 ± 1.6 % of the radioactivity was still associated with unmetabolized radiotracer (Fig. 4b, and Suppl. Figs. 3 and 4).

## Discussion

[^18^F]Selectfluor bis(triflate) can be easily produced from [^18^F]F_2_ by bubbling it through a solution of the adequate azoniabicyclo[2.2.2]octane triflate precursor. In addition to being a milder electrophilic fluorination reagent, the use [^18^F]Selectfluor bis(triflate) can allow for a longer reaction time compared to direct [^18^F]F_2_ labeling, as well as heating and stirring of the reaction mixture. A major advantage of using [^18^F]Selectfluor bis(triflate) is also that this reagent is compatible with temporary storage, and as such, crude stocks can easily be split into smaller portions, allowing multiple reactions to be carried out from a single batch of cyclotron-produced fluorine-18. Additionally, such crude stocks can also be used without further purification since the unlabeled Selectfluor bis(triflate) precursor and [^18^F]LiF both present in the reagent solution do not interfere with subsequent labeling reactions.

With respect to the use of [^18^F]Selectfluor bis(triflate), a comparison of entries 1, 3, and 5 (Table 1) with 2, 4, and 6, respectively, demonstrates that a longer reaction time did not afford a higher conversion. Hence, following the initial test conditions, a reaction time of 15 min and a double concentration of the reagents were the conditions chosen for the production of [^18^F]F-DPA batches for preclinical evaluation.

When [^18^F]F_2_ was employed as the [^18^F]fluorinating reagent, multiple fluorinated products were observed. Interestingly, none of the radiolabeled products were missing the tributyl tin moiety. This finding shows that elemental fluorine is far too reactive to carry out selective fluorination on this relatively structurally complex derivative. The varying degrees of fluorination observed suggest that there are multiple sites within the F-DPA precursor molecule (compound **1** in Fig. 1) that are susceptible to fluorination by elemental fluorine gas. These may include *ortho* and *meta* positions of the *para*-substituted phenyl ring as well as the amide moiety.

The radiometabolite analyses of plasma and brain homogenates showed that [^18^F]F-DPA is more metabolically stable than [^18^F]DPA-714 in rats. The *R*_f_ values for radiometabolites of [^18^F]F-DPA are only slightly lower than of the parent molecule, thereby suggesting that while these metabolites are slightly more polar than [^18^F]F-DPA, they may be structurally similar to the injected radiotracer. In the case of [^18^F]DPA-714, the major metabolite appears at the plate origin thereby suggesting that it is strongly different in structure from the parent molecule. This metabolite in all likelihood results from the cleavage of the fluoroethoxy chain which generates [^18^F]fluoroacetaldehyde and subsequently [^18^F]fluoroacetate as previously described by Peyronneau *et al*. [21]. While [^18^F]F-DPA is only slightly more metabolically stable than [^18^F]DPA-714 in rat plasma (Fig. 4a), in the brain, unmetabolized [^18^F]F-DPA accounts for the majority of the remaining radioactivity at 90 min after injection (Fig. 4b). This result suggests that the major radiometabolites of [^18^F]F-DPA only poorly cross the blood-brain barrier, and hence, should not contribute to non-specific signals. While there is somewhat of a difference between the metabolic profile of [^18^F]DPA-714 reported here and with previously reported data [21, 29], this difference may be explained by the differing analytical methods used. While sometimes considered a less sensitive analytical method, radioTLC allows the visualization of entire radioactivity content of the sample, while radioHPLC will only detect those radioactive products that elute from column. Furthermore, the short time required to perform radioTLC and the possibility to run multiple samples simultaneously make it an attractive analytical method for the sensitive analyses of low radioactivity samples.

While there is a large difference between the free and plasma bound radioactivity fractions for [^18^F]F-DPA and [^18^F]DPA-714 at 15 min after injection of the tracer, this large difference between the two tracers can be explained in terms of their respective metabolic routes. [^18^F]DPA-714 is metabolized primarily by cleavage of the fluorine-18-containing side chain [21]. [^18^F]F-DPA, on the other hand, does not contain the fluorine-18-labeled side chain; hence, the metabolism will give radiometabolites that are more structurally similar to the parent molecule. These radiometabolites would then bind to the plasma proteins in a similar way as the unchanged [^18^F]F-DPA.

The fast clearance of non-specific uptake of radioactivity in the brain also demonstrates that radiotracer concentration equilibrium is reached rapidly. This is a favorable characteristic since it means that a PET image of the rat brain can be acquired 20–30 min after radiotracer injection. In addition, when this result is combined with the findings of the radiometabolite analysis of brain homogenate, it can be seen that during this period, the percentage of radioactivity of the unchanged tracer is greater than 95 %. Hence, a PET image acquired during this period will correspond to the specifically bound tracer and will not suffer from the drawback of background signal resulting from non-specifically bound radioactivity. The lack of increased accumulation of radioactivity as a function of time in the bone of the skull, indicating little defluorination of the tracer, is a pleasing observation as it means that the PET images of the brain will not be affected by spillover from the signal in the bone.

While the results presented in this manuscript do not directly address the issue of specificity of [^18^F]F-FDA binding to TSPO in neuroinflammation, i.e., no competitive blockade study was performed in an animal model with neuroinflammation, a high uptake of [^18^F]F-FDA as well as [^18^F]F-FDA-714 can be seen, as expected, in steroid-producing TSPO rich organs such as the adrenals, liver, and kidneys in rat (see Suppl. Table 1), thereby, suggesting the specificity of [^18^F]F-FDA *in vivo*. Furthermore, both the affinity (*K*_i_) and selectivity (*K*_i_ CBR) of the noval tracer (*K*_i_ = 1.7 nM, *K*_i_ CBR > 1 μM)[25] are comparable with those of the routinely used [^18^F]F-FDA-174 [17] (*K*_i_ = 0.91 ± 0.08 nM, *K*_i_ CBR > 1 μM) [24, 30, 31]. When this is combined with the specific binding shown for [^18^F]F-FDA-174 [17], it gives promise to [^18^F]F-FDA as a new TSPO ligand.

## Conclusions

[^18^F]Selectfluor bis(triflate) was successfully used to introduce fluorine-18 at the *para*-position of a 2-phenyl-pyrazolo[1,5a]pyrimidine motif, leading to the synthesis of [^18^F]F-DPA. As demonstrated herein, the binding of the fluorine-18 atom directly to the aromatic ring is more favorable with respect to *in vivo* metabolism and [^18^F]fluoride release, when compared to the fluoroalkoxy derivative [^18^F]DPA-714, where the fluorine-18 atom is bound to the aromatic ring via the alkoxy linker.

Due to the different labeling strategies adopted, there is a greater than 100-fold difference between the SAs of the two radiotracers (even though [^18^F]F-DPA is on the higher end of the scale for SA achieved by electrophilic fluorination). Despite this difference, the quality of the PET-data obtained *in vivo* suggests that high SA is not a critical factor when using a TSPO ligand belonging to the pyrazolopyrimidine acetamide class. While a low SA may pose a problem in terms of receptor occupancy for not very abundant receptors, TSPO is an abundant target in neuroinflammation and a sensitive indirect index of neuronal damage [32] that is expressed throughout the whole brain, and hence, there should not be an issue with the receptor occupancy in terms of competition between the radioactively labeled and unlabeled analogues, at least, in cases of high overexpression of TSPO.

The biodistributions of [^18^F]F-DPA and [^18^F]DPA-714 compare well to one another and the major difference between these radiotracers stems mainly from their respective metabolic profiles. Due to fast washout of radioactivity, resulting in lower background signal of non-specifically distributed radiometabolites and the high proportion of unchanged [^18^F]F-DPA in the rat brain, this novel radiotracer can provide better target-to-background ratios than [^18^F]DPA-714. The results of this study show that [^18^F]F-DPA is a promising TSPO radiotracer, and hence, warrants further investigation in animal models of disease.

## Electronic Supplementary Material

Below is the link to the electronic supplementary material.

ESM 1 (PDF 1029 kb)

## References

[1] Anholt R, Pedersen P, De Souza E, Snyder S (1986). The peripheral-type benzodiazepine receptor. Localization to the mitochondrialouter membrane. J Biol Chem.

[2] Papadopoulos V, Amri H, Boujrad N (1997). Peripheral benzodiazepine receptor in cholesterol transport and steroidogenesis. Steroids.

[3] Banati R (2002). Visualising microglial activation *in vivo*. Glia.

[4] Dollé F, Luus C, Reynolds A, Kassiou M (2009). Radiolabelled molecules for imaging the translocator protein (18 kDa) using positron emission tomography. Curr Med Chem.

[5] O’Brien E, Kersemans V, Tredwell M (2014). Glial activation in the early stages of brain metastasis: TSPO as a diagnostic biomarker. J Nucl Med.

[6] Inoue O, Yamasaki T, Hashimoto K, Kojima M (1985). Evaluation of 3H-PK11195 as a radioligand for the *in vivo* study of peripheral benzodiazepine receptor. Kaku Igaku.

[7] Camsonne R, Moulin MA, Crouzel C (1986). C-11 labeling of PK11195 and visualization of peripheral receptors of benzodiazepines by positron-emission tomography. J Pharmacol.

[8] Watkins GL, Jewett DM, Mulholland GK (1988). A captive solvent method for rapid N-[^11^C]methylation of secondary amides: application to the benzodiazepine, 4′-chlorodiazepam (RO5-4864). Int J Rad Appl Instrum A.

[9] Gulyás B, Halldin C, Karlsson P (1999). Brain uptake and metabolism of [^11^C]vinpocetine. J Neuroimaging.

[10] Zhang MR, Kumata K, Maeda J (2007). ^11^C-AC-5216: a novel PET ligand for peripheral benzodiazepine receptors in the primate brain. J Nucl Med.

[11] Briard E, Zoghbi SS, Imaizumi M (2008). Synthesis and evaluation in monkey of two sensitive ^11^C-labeled aryloxyanilide ligands for imaging brain peripheral benzodiazepine receptors *in vivo*. J Med Chem.

[12] Briard E, Hong J, Musachio JL (2005). Synthesis and evaluation of two candidate ^11^C-labeled radioligands for brain peripheral benzodiazepine receptors [abstract]. J Label Compd Radiopharm.

[13] Wadsworth H, Jones PA, Chau WF (2012). [^18^F]GE-180: a novel fluorine-18 labelled PET tracer for imaging translocator protein 18 kDa (TSPO). Bioorg Med Chem Lett.

[14] Thominiaux C, Damont A, Kuhnast B (2010). Radiosynthesis of 7-chloro-N, N-dimethyl-5-[11C]methyl-4-oxo-3-phenyl-3,5-dihydro-4H-pyridazino[4,5-b]indole-1-acetamide, [11C]SSR180575, a novel radioligand for imaging the TSPO (peripheral benzodiazepine receptor) with PET. J Label Compounds Radiopharm.

[15] Tiwari AK, Fujinaga M, Yui J (2014). Synthesis and evaluation of new ^18^F-labelled acetamidobenzoxazolone-based radioligands for imaging of the translocator protein (18 kDa, TSPO) in the brain. Org Biomol Chem.

[16] James ML, Fulton RR, Hendreson DJ (2005). Synthesis and *in vivo* evaluation of a novel peripheral benzodiazepine receptor PET radioligand. Bioorg Med Chem.

[17] James ML, Fulton RR, Vercoullie J (2008). DPA-714, a new translocator protein-specific ligand: Synthesis, radiofluorination, and pharmacologic characterization. J Nucl Med.

[18] Fookes C, Pham T, Mattner F (2008). Synthesis and biological evaluation of substituted [^18^F]imidazo[1,2-a]pyridines and [^18^F]pyrazolo[1,5-a]pyrimidines for study of peripheral benzodiazepine receptor using positron emission tomography. J Med Chem.

[19] Boutin H, Chauveau F, Thominiaux C (2007). *In vivo* imaging of brain lesions with [^11^C]CLINME, a new PET radioligand of peripheral benzodiazepine receptors. Glia.

[20] Katsifis A, Fookes C, Pham T, et al (2007) Fluorinated ligands for targeting peripheral benzodiazepine receptors. AUS Patent 2006904617

[21] Peyronneau MA, Saba W, Goutal S (2013). Metabolism and quantification of [^18^F]DPA-714, a new TSPO positron emission tomography radioligand. Drug Metab Dispos.

[22] Peyronneau M, Loc’h C, Mattner F (2011). *In vitro* metabolism of 18F PBR102 and 18F PBR111 in rats and humans. Eur J Nucl Med Mol Imaging.

[23] Banister SD, Shen B, Chin FT, Kassiou M (2014) Metabolically fortified DPA-714 analogs for improved PET imaging of translocator protein (TSPO) [abstract]. Abstr Pap Am Chem S 248:219-MEDI

[24] Damont A, Médran-Navarrete V, Cacheux F (2015). Novel pyrazolo[1,5-a]pyrimidines as translocator protein 18 Da (TSPO) ligands: synthesis, *in vitro* biological evaluation, [18F]-labeling, and *in vivo* neuroinflammation PET images. J Med Chem.

[25] Damont A, Caillé F, Medran-Navarrete V (2015). The pyrazolo[1,5-a]pyrimidine F-DPA: synthesis, *in vitro* characterization and radiolabeling with fluorine-18 using a nucleophilic approach [abstract]. J Label Compd Radiopharm.

[26] Teare H, Robins EG, Kirjavainen AK (2010). Radiosynthesis and evaluation of [^18^F]Selectfluor bis(triflate). Angew Chem Int Ed Engl.

[27] Damont A, Hinnen F, Kuhnast B (2008). Radiosynthesis of [^18^F]DPA-714, a selective radioligand for imaging the translocator protein (18 kDa) with PET. J Label Compd Radiopharm.

[28] Bergman J, Solin O (1997). Fluorine-18-labeled fluorine gas for synthesis of tracer molecules. Nucl Med Biol.

[29] Chauveau F, Van Camp N, Dollé F (2009). Comparative evaluation of the translocator protein radioligands 11CDPA-713, 18F-DPA-714, and 11C-PK11195 in a rat model of acute neuroinflammation. J Nucl Med.

[30] Medran-Navarrete V, Damont A, Peyronneau M (2014). Preparation and evaluation of novel pyrazolo[1,5-a]pyrimidine acetamides, closely related to DPA-714, as potent ligands for imaging the TSPO 18 kDa with PET. Bioorg Med Chem Lett.

[31] Cacheux F, Medran-Navarrete V, Dolle F (2017). Synthesis and *in vitro* characterization of novel fluorinated derivatives of the translocator protein 18 kDa ligand CfO-DPA-714. Eur J Med Chem.

[32] Benavides J, Fage D, Carter C (1987). Peripheral type benzodiazepine binding sites are a sensitive indirect index of neuronal damage. Brain Res.

