# Supplementary material for: Radiosynthesis and Preclinical Evaluation of [18F]F-DPA, A Novel Pyrazolo[1,5a]pyrimidine Acetamide TSPO Radioligand, in Healthy Sprague Dawley Rats
Source: Mol Imaging Biol. 2017 Jan 12;19(5):736–45. doi: 10.1007/s11307-016-1040-z (PMC5574958; doi:10.1007/s11307-016-1040-z)
Supplement: Supplementary file 1 — (PDF 1029 kb) [file 11307_2016_1040_MOESM1_ESM.pdf]

Electronic Supplementary Material

Radiosynthesis and Preclinical Evaluation of $[^{18}F]$F-DPA, a Novel Pyrazolo[1,5a]pyrimidine Acetamide TSPO Radioligand

Journal: Molecular Imaging and Biology

Keller Thomas[1], Krzyczmonik Anna[1], Forsback Sarita[1,2], Lopez Picon Francisco[3,4], Kirjavainen Anna[1], Almajidi Rana[3,4], Takkinen Jatta[3,4], Rajander Johan[5], Cacheux Fanny[6], Damont Annelaure[6], Dollé Frédéric[6], Rinne Juha O[7], Haaparanta-Solin Merja[3,4], Solin Olof[1,2,5]

1 Radiopharmaceutical Chemistry Laboratory, Turku PET Centre, University of Turku, Finland

2 Department of Chemistry, University of Turku, Finland

3 PET Preclinical Imaging Laboratory, Turku PET Centre, University of Turku, Finland

4 MediCity Research Laboratory, University of Turku, Finland

5 Accelerator Laboratory, Turku PET Centre, Åbo Akademi University, Finland

6 CEA, I2BM, Service Hospitalier Frédéric Joliot, Orsay, France

7 Turku PET Centre, Turku University Hospital, Finland

Correspondence: Prof. Olof Solin, Turku PET Centre, University of Turku, Kiinamyllynkatu 4-8, FI-20520 Turku, Finland, Fax: +358 2 2318191, Tel: +358 2 3132851, Email: olof.solin@abo.fi

	%ID/g tissue									
	5 min		15 min		30 min		60 min		90 min	
	[18F]F-DPA	[18F]DPA-714	[18F]F-DPA	[18F]DPA-714	[18F]F-DPA	[18F]DPA-714	[18F]F-DPA	[18F]DPA-714	[18F]F-DPA	[18F]DPA-714
Blood	0.12 ± 0.01	0.37 ± 0.03	0.11 ± 0.02	0.19 ± 0.005	0.07 ± 0.006	0.11 ± 0.004	0.05 ± 0.004	0.09 ± 0.004	0.05 ± 0.001	0.08 ± 0.002
Plasma	0.03 ± 0.002	0.20 ± 0.05	0.03 ± 0.001	0.05 ± 0.02	0.03 ± 0.004	0.04 ± 0.008	0.03 ± 0.005	0.06 ± 0.005	0.02 ± 0.004	0.06 ± 0.004
Erythrocytes	0.22 ± 0.03	0.56 ± 0.008	0.19 ± 0.05	0.35 ± 0.03	0.11 ± 0.01	0.18 ± 0.02	0.08 ± 0.006	0.13 ± 0.009	0.08 ± 0.007	0.112 ± 0.005
Adrenal x2	3.64 ± 1.01	2.45 ± 0.44	4.74 ± 1.46	6.17 ± 0.94	5.64 ± 1.97	4.46 ± 0.90	6.11 ± 1.21	5.58 ± 1.37	5.63 ± 2.06	6.77 ± 2.88
Thymus	0.81 ± 0.12	0.54 ± 0.006	0.95 ± 0.05	0.62 ± 0.05	0.97 ± 0.04	0.60 ± 0.04	1.08 ± 0.06	0.63 ± 0.05	0.97 ± 0.08	0.59 ± 0.03
Liver, sample	0.73 ± 0.26	0.65 ± 0.14	0.88 ± 0.14	0.86 ± 0.20	0.52 ± 0.15	0.59 ± 0.01	0.45 ± 0.08	0.35 ± 0.01	0.34 ± 0.06	0.27 ± 0.03
Spleen	2.35 ± 0.67	3.45 ± 0.80	3.64 ± 0.72	3.64 ± 1.09	2.51 ± 0.40	3.60 ± 0.16	2.38 ± 0.24	3.14 ± 0.10	1.96 ± 0.17	2.67 ± 0.22
Pancreas	1.39 ± 0.60	0.96 ± 0.10	1.06 ± 0.07	1.41 ± 0.32	0.81 ± 0.14	1.05 ± 0.18	0.59 ± 0.12	1.01 ± 0.04	0.49 ± 0.04	0.84 ± 0.03
Heart	5.90 ± 1.93	3.38 ± 0.64	6.16 ± 0.62	4.22 ± 0.44	4.84 ± 0.65	4.73 ± 1.21	3.01 ± 0.77	3.84 ± 0.71	2.68 ± 0.77	3.28 ± 0.29
Kidney x2	3.72 ± 0.30	3.11 ± 0.28	3.73 ± 0.32	3.27 ± 0.50	2.75 ± 0.04	3.21 ± 0.27	2.31 ± 0.19	2.57 ± 0.04	1.91 ± 0.08	2.28 ± 0.15
Fat sub	0.08 ± 0.008	0.08 ± 0.02	0.15 ± 0.07	0.11 ± 0.03	0.11 ± 0.04	0.13 ± 0.04	0.23 ± 0.03	0.29 ± 0.07	0.24 ± 0.06	0.37 ± 0.02
Urine + bladder	0.14 ± 0.02	0.06 ± 0.02	0.15 ± 0.03	0.10 ± 0.007	0.37 ± 0.16	0.31 ± 0.08	0.45 ± 0.07	0.25 ± 0.07	0.34 ± 0.02	0.32 ± 0.07
Bone (from skull)	0.25 ± 0.11	0.18 ± 0.09	0.30 ± 0.07	0.21 ± 0.08	0.26 ± 0.06	0.27 ± 0.02	0.24 ± 0.09	0.25 ± 0.14	0.25 ± 0.04	0.28 ± 0.03

Muscle	0.07 ± 0.008	0.07 ± 0.005	0.07 ± 0.001	0.07 ± 0.03	0.09 ± 0.01	0.11 ± 0.04	0.11 ± 0.03	0.16 ± 0.03	0.12 ± 0.03	0.18 ± 0.04
Testis	0.21 ± 0.07	0.11 ± 0.03	0.28 ± 0.02	0.10 ± 0.001	0.23 ± 0.03	0.13 ± 0.03	0.26 ± 0.07	0.14 ± 0.04	0.27 ± 0.05	0.14 ± 0.01
Lung	5.94 ± 0.13	19.83 ± 3.18	4.04 ± 1.58	10.24 ± 0.48	2.27 ± 0.07	5.73 ± 0.50	1.79 ± 0.31	3.01 ± 0.21	1.47 ± 0.12	2.00 ± 0.03
Salivary x2	0.70 ± 0.13	0.59 ± 0.06	0.82 ± 0.14	1.34 ± 0.46	1.56 ± 0.40	0.88 ± 0.20	1.21 ± 0.17	1.00 ± 0.06	1.00 ± 0.13	0.89 ± 0.09
Eye x2	0.32 ± 0.05	0.28 ± 0.02	0.29 ± 0.08	0.29 ± 0.04	0.21 ± 0.03	0.26 ± 0.05	0.16 ± 0.02	0.22 ± 0.04	0.16 ± 0.04	0.20 ± 0.02
Brown fat	0.28 ± 0.09	0.22 ± 0.03	0.43 ± 0.06	0.31 ± 0.14	1.11 ± 1.15	0.51 ± 0.08	0.44 ± 0.07	1.07 ± 0.68	0.95 ± 0.65	1.25 ± 0.04
Harderian gland	0.54 ± 0.06	0.40 ± 0.02	0.59 ± 0.09	0.52 ± 0.05	0.62 ± 0.02	0.45 ± 0.02	0.83 ± 0.21	0.54 ± 0.02	0.55 ± 0.11	0.43 ± 0.03
Brain	0.23 ± 0.03	0.29 ± 0.04	0.17 ± 0.05	0.17 ± 0.004	0.09 ± 0.009	0.12 ± 0.01	0.07 ± 0.008	0.09 ± 0.003	0.06 ± 0.006	0.08 ± 0.005
Liver	0.77 ± 0.15	0.78 ± 0.19	0.82 ± 0.10	0.87 ± 0.12	0.49 ± 0.13	0.60 ± 0.04	0.43 ± 0.05	0.37 ± 0.02	0.31 ± 0.05	0.26 ± 0.04
Injection site (tail)	0.36 ± 0.03	0.49 ± 0.43	0.38 ± 0.10	0.29 ± 0.02	0.28 ± 0.008	0.34 ± 0.07	0.42 ± 0.21	0.38 ± 0.07	0.38 ± 0.07	0.24 ± 0.06
Stomach wall + contents	0.50 ± 0.15	0.27 ± 0.11	0.54 ± 0.18	0.65 ± 0.20	0.93 ± 0.16	0.43 ± 0.23	1.07 ± 0.09	0.33 ± 0.05	0.74 ± 0.23	0.44 ± 0.15
Small int. wall + contents	1.19 ± 0.09	0.69 ± 0.13	1.47 ± 0.18	1.07 ± 0.11	2.00 ± 0.54	1.44 ± 0.19	1.95 ± 0.23	1.80 ± 0.30	2.59 ± 0.19	2.04 ± 0.24
Large int. Wall + contents	0.30 ± 0.02	0.20 ± 0.04	0.31 ± 0.03	0.24 ± 0.008	0.33 ± 0.05	0.26 ± 0.02	0.29 ± 0.02	0.41 ± 0.23	0.28 ± 0.02	0.29 ± 0.02

Supplementary Table 1. Uptake of fluorine-18-radioactivity in organs of interest of Sprague Dawley rats, at various time points after injection of [18F]F-DPA or [18F]DPA-714. Values are expressed as %ID/g tissue (means ± SD, n = 3)

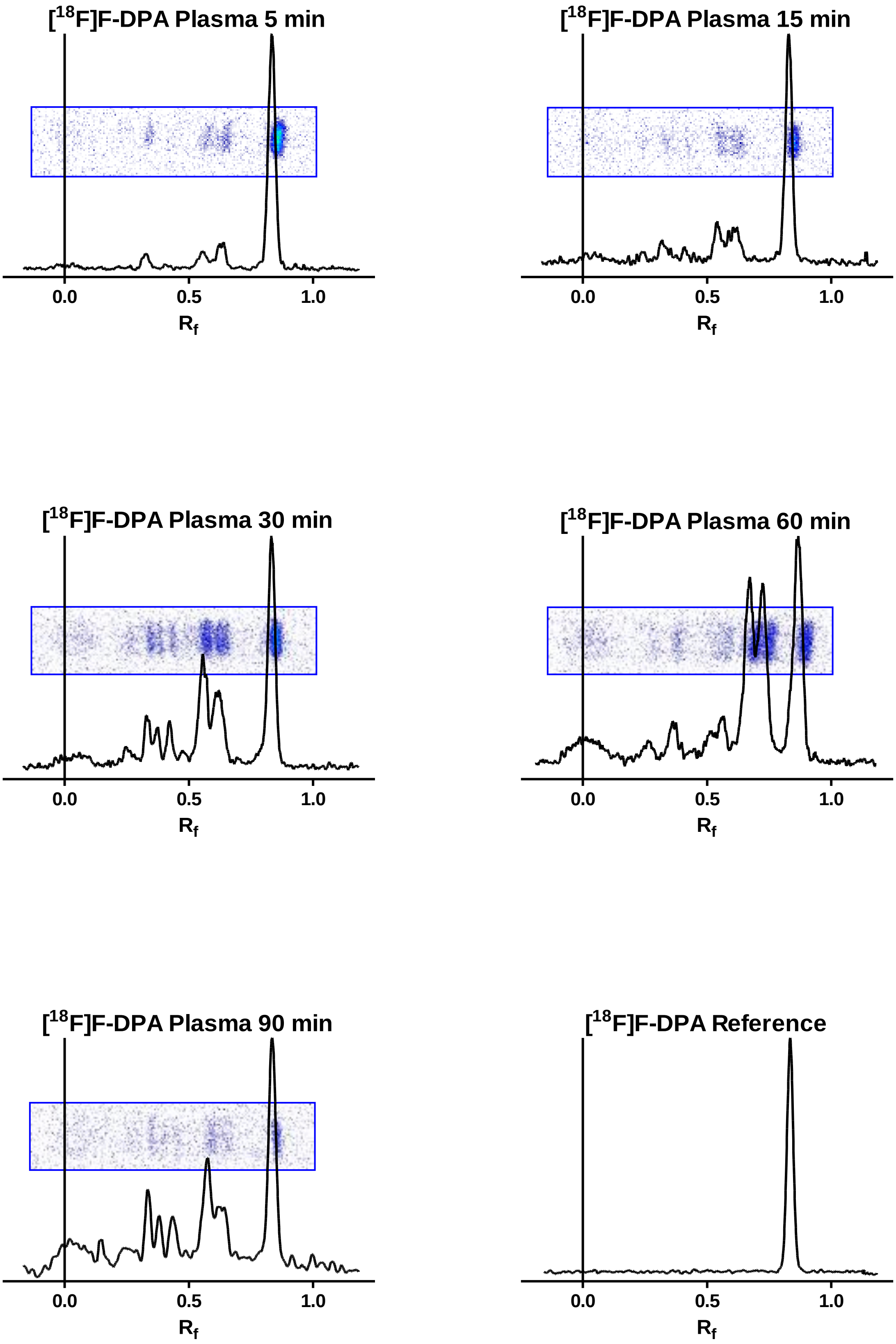

Supplementary Figure 1. TLC autoradiograms of plasma of Sprague Dawley rats, following 5, 15, 30, 60 and 90 min injection of [^{18}F]F-DPA, and reference [^{18}F]F-DPA.

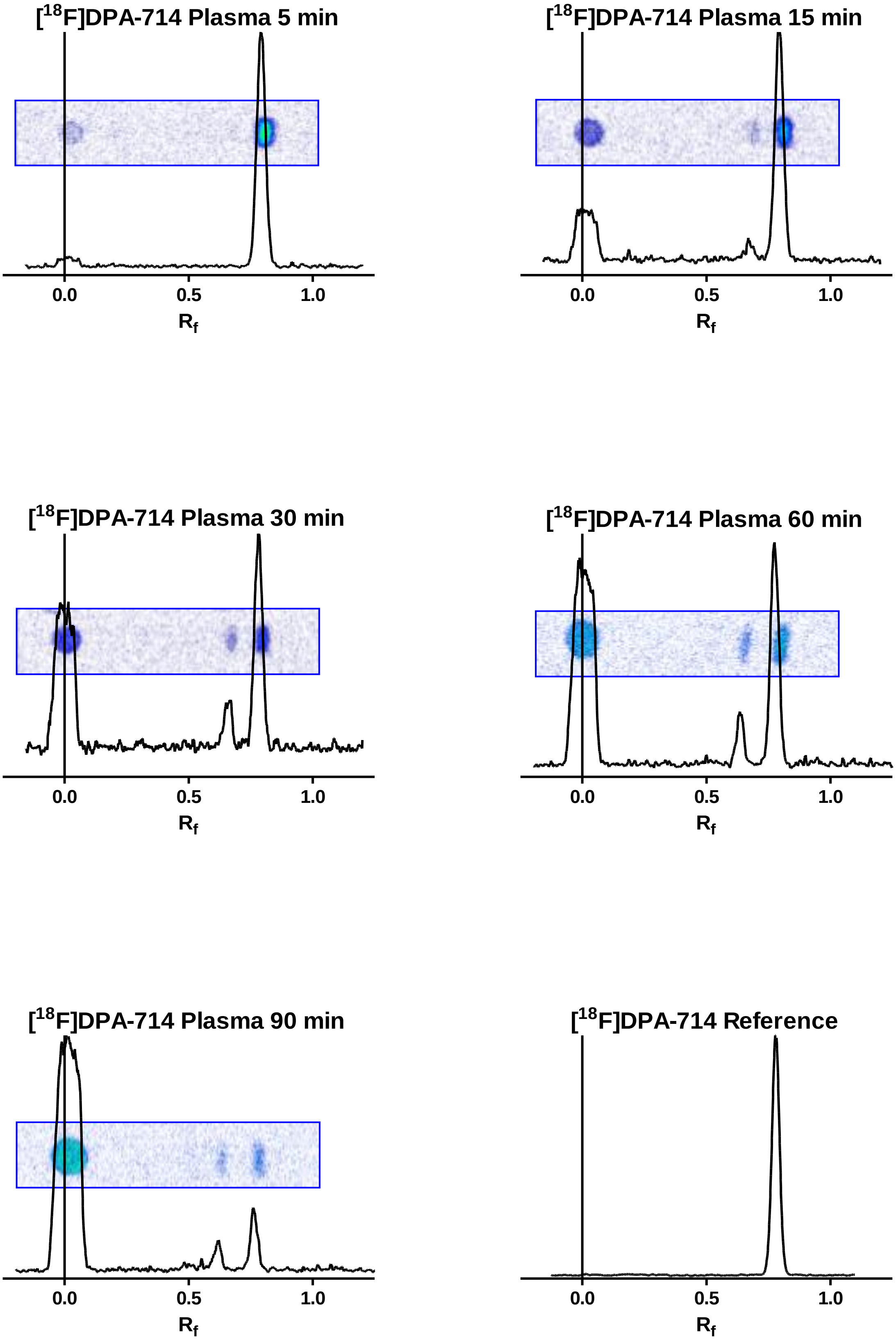

Supplementary Figure 2. TLC autoradiograms of plasma of Sprague Dawley rats, following 5, 15, 30, 60 and 90 min injection of [18F]DPA-714, and reference [18F]DPA-714.

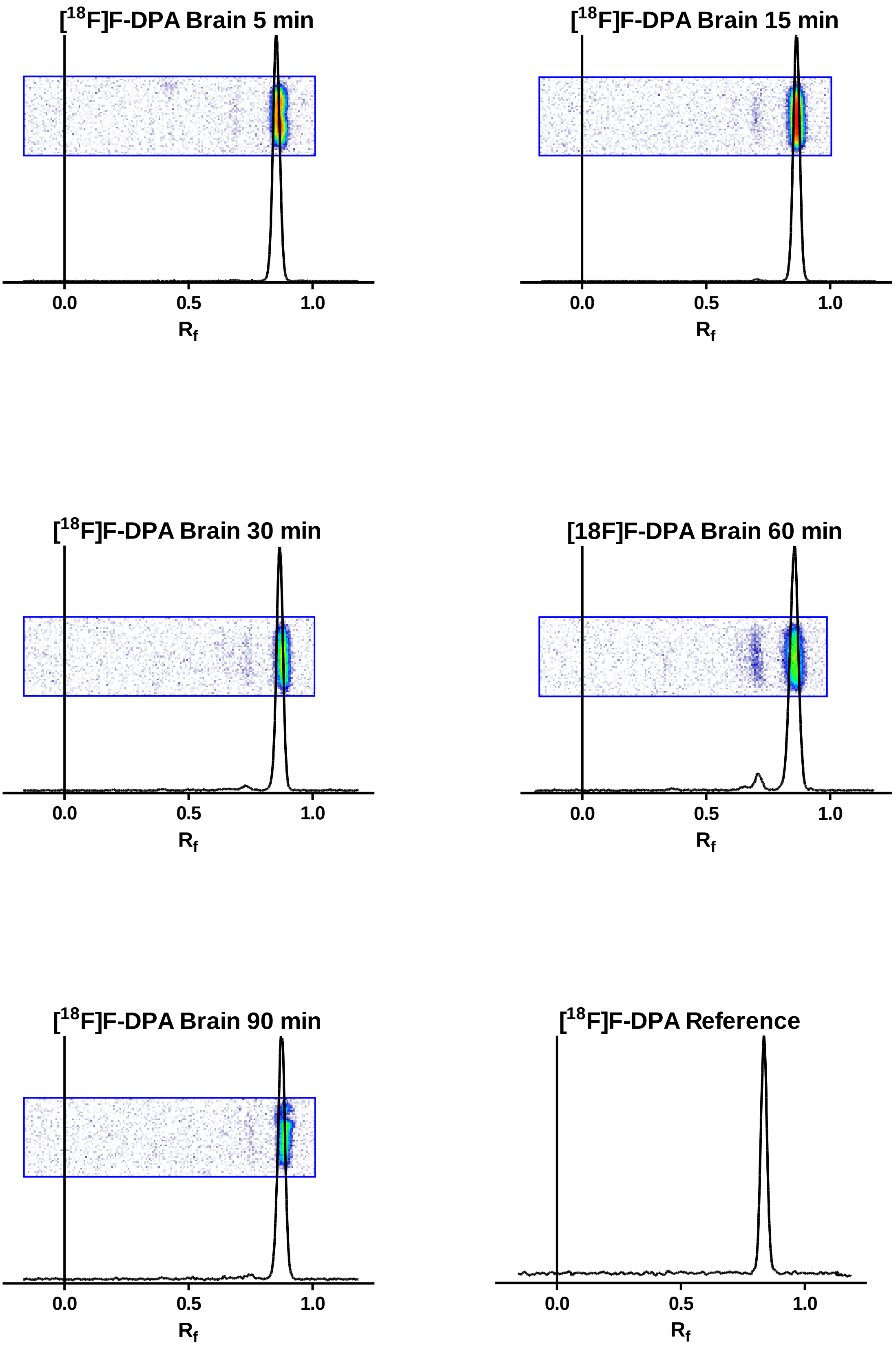

Supplementary Figure 3. TLC autoradiograms of brain homogenate of Sprague Dawley rats, following 5, 15, 30, 60 and 90 min injection of [^{18}F]F-DPA, and reference [^{18}F]F-DPA.

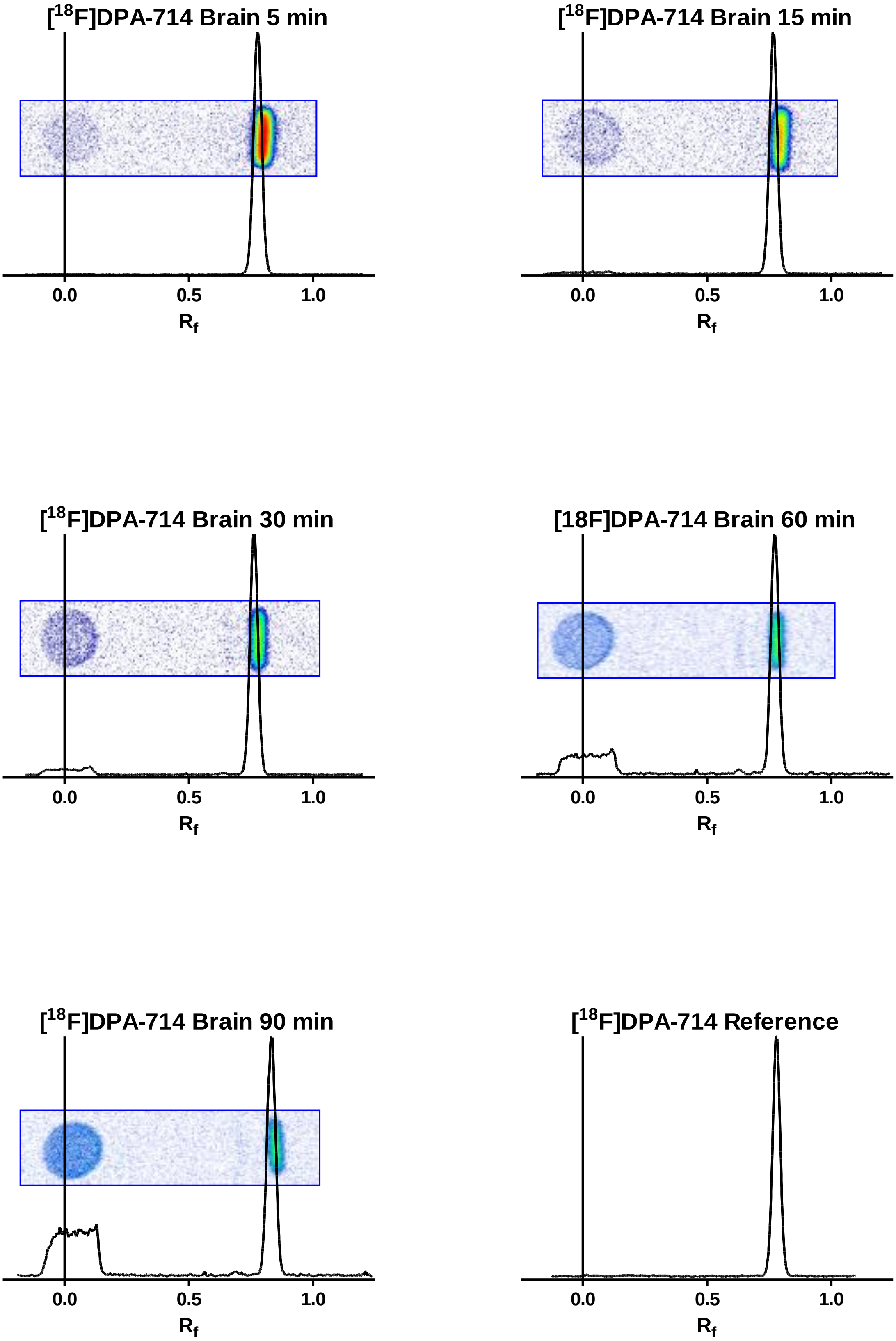

Supplementary Figure 4. TLC autoradiograms of brain homogenate of Sprague Dawley rats, following 5, 15, 30, 60 and 90 min injection of [^{18}F]DPA-714, and reference [^{18}F]DPA-714.

Supplementary Materials and Methods

Chemicals

All organic solvents were HPLC-grade and purchased from Sigma-Aldrich (Steinheim, Germany). Potassium carbonate and acetone-d_6 were also purchased from Sigma-Aldrich. Kryptofix 222 (4,7,13,16,21,24-hexaoxa-1,10-diazabicyclo[8.8.8]hexacosane, K_{222}) and sodium dihydrogen phosphate were purchased from Merck KGaA (Darmstadt, Germany). Ammonium acetate was bought from Alfa Aesar (Karlsruhe, Germany). Ethanol and saline (0.9% aq. NaCl) for formulation were purchased from Berner Oy (Helsinki, Finland) and B. Braun Medical Oy (Helsinki, Finland). Oxygen-18-enriched Hyox water, for fluorine-18-production, was purchased from Rotem Industries Ltd. (Arava, Israel). All gases were supplied by AGA, Linde group (Espoo, Finland).

HPLC

Preparative HPLC was performed using either a Merck Hitachi L-6200 pump with a L-7400 UV detector and a NaI(TI) scintillator detector or a Jasco PU-2089 pump and a UV detector. HPLC analysis was carried out using either a Merck Hitachi LaChrom 7000 system with Merck Hitachi D-7000 HPLC System Manager software (version 3.1.1), or a VWR Hitachi LaChrom Elite system with EXChrom Elite Client / Server software (version 3.1.6). Analyses were carried out with a wavelength of 280 nm and the radioactivity was detected with a NaI(TI) scintillator detector.

N,N-Diethyl-2-(2-(4-fluorophenyl)-5,7-dimethylpyrazolo[1,5-a]pyrimidin-3-yl)acetamide (F-DPA, Fig. 1a, compound 2)

R_f (dichloromethane/methanol: 93/7 v/v): 0.46. ^{1}H-NMR ($CDCl_3$, 400 MHz): δ 7.82 (dd, 2H, J^3_{HH} = 8.4 Hz, J^3_{HF} = 5.6 Hz, Ph), 7.13 (t, 2H, J = 8.4 Hz, Ph), 6.52 (s, 1H), 3.91 (s, 2H, CH_2), 3.51 (q, 2H, J = 7.2 Hz, NCH_2CH_3), 3.40 (q, 2H, J = 7.2 Hz, NCH_2CH_3), 2.73 (s, 3H, CH_3), 2.54 (s, 3H, CH_3), 1.21 (t, 3H, J = 7.2 Hz, NCH_2CH_3), 1.10 (t, 3H, J = 7.2 Hz, NCH_2CH_3). ^{13}C-NMR ($CDCl_3$, 100 MHz): δ 169.8 [C], 164.2 [C], 161.8 [C], 157.6 [C], 154.6 [C], 146.1

[d, J^1_{CF} = 130 Hz, C], 130.5 [d, J^3_{CF} = 8 Hz, 2xCH], 129.6 [C], 115.4 [d, J^2_{CF} = 22 Hz, 2xCH], 108.2 [CH], 101.1 [C], 42.3 [CH_2], 40.6 [CH_2], 27.9 [CH_2], 24.1 [CH_3], 16.8 [CH_3], 14.2 [CH_3], 13.0 [CH_3]. MS (ESI+) m/z 355 $[M+H]^+$.

N,N-Diethyl-2-(2-(4-(tributylstannyl)phenyl)-5,7-dimethyl-pyrazolo[1,5-α]pyrimidin-3-yl)acetamide (F-DPA labeling precursor, Fig. 1a, compound 1).

To a suspension of *N,N*-diethyl-2-(2-(4-iodophenyl)-5,7-dimethylpyrazolo[1,5-*a*]pyrimidin-3-yl) acetamide (I-DPA synthesized according to Damont *et al.* [25]:, 258 mg, 0.56 mmol) in *N,N*-dimethylformamide (10 ml) under argon, were added *tetra*kis(triphenylphosphine)palladium (64.4 mg, 0.06 mmol) and hexabutylditin (647.44 mg, 1.12 mmol). The reaction mixture was stirred overnight at 120 °C. After cooling to room temperature, the mixture was partitioned between water (50 ml) and ethyl acetate (50 ml). The aqueous layer was separated and extracted with ethyl acetate (3 x 50 ml). The combined organic layers were washed with brine (100 ml), dried over Na_2SO_4 and concentrated to dryness under vacuum. The crude product was purified by flash chromatography on silica gel (heptane/ethyl acetate: 90/10 to 80/20 then, toluene/acetone/triethylamine: 95/5/0.001 to 90/10/0.001) to afford the desired compound (113 mg, 32 %) as a white solid.

R_f (toluene/acetone: 80/20): 0.12. ^{1}H-NMR ($CDCl_3$, 400 MHz): δ 7.76 (d, 2H, J = 12.0 Hz), 7.57 (d, 2H, J^3_{HH} = 12.0 Hz), 6.51 (s, 1H), 3.93 (s, 2H), 3.49 (q, 2H, J^3_{HH} = 7.2 Hz), 3.42 (q, 2H, J^3_{HH} = 7.2 Hz), 2.74 (s, 3H), 2.54 (s, 3H), 1.58 - 1.50 (m, 6 H), 1.38 - 1.28 (m, 6H), 1.20 (t, 3H, J^3_{HH} = 7.2 Hz), 1.10 (t, 3H, J^3_{HH} = 7.2 Hz), 1.06 - 0.99 (m, 6H), 0.88 (t, 9H, J^3_{HH} = 7.2 Hz). ^{13}C-NMR ($CDCl_3$, 100 MHz): δ 174.6 [C], 170 [C], 157.4 [C], 155.2 [C], 144.7 [C], 142.15 [C], 136.5 [2CH], 133.1 [C], 128.1 [2CH], 108.2 [CH], 101.1 [C], 42.2 [CH_2], 40.5 [CH_2], 29 [3xCH_2], 27.7 [CH_2], 26.7 [3xCH_2], 24.5 [CH_3], 16.8 [CH_3], 14.2 [CH_3], 13.6 [3xCH_2], 13 [CH_3], 7.8 [3xCH_3].

Radionuclide Production

Fluorine-18 was produced as [^{18}F]fluoride via the $^{18}O(p,n)^{18}F$ nuclear reaction by either a CC-18/9 cyclotron (Efremov Scientific Institute of Electrophysical Apparatus, St. Petersburg, Russia) or an MGC-20 cyclotron

(Efremov Scientific Institute of Electrophysical Apparatus, Leningrad, Russia). Using the CC-18/9 cyclotron, a niobium target containing 2.3 ml of ^{18}O-18 enriched water was irradiated with a 17 MeV proton beam. The target content was then passed over an anion exchange cartridge (QMA Sep Pak, Waters Corporation, Milford, MA, USA) and the aqueous [^{18}F]fluoride was transported to the reaction vessel. Using the MGC-20 cyclotron, a silver target containing only 800 µl of O-18 enriched water was employed and irradiated with a 17 MeV proton beam. In this case, the target content (i.e., the irradiated water containing [^{18}F]fluoride) was transported directly to the reaction vessel without being passed over a QMA cartridge. [^{18}F]Fluoride used for initial developmental reactions was produced using the CC-18/9 cyclotron whereas [^{18}F]F-DPA batches for preclinical evaluation used [^{18}F]fluoride produced by the MGC-20 cyclotron.

[^{18}F]F_2 synthesis

Aqueous [^{18}F]fluoride from the cyclotron-target was added to a reaction vessel containing Kryptofix 222 (24.3 ± 2.3 mg, 64.5 ± 6.1 µmol) and K_2CO_3 (6.9 ± 0.7 mg, 50.0 ± 4.8 µmol). CH_3CN (1 ml) was then added to the vessel and the solvents were removed by azeotropic distillation, carried out for 4 min at a temperature of 100 °C under a helium flow. Two further additions of CH_3CN (1 ml each) were made, each followed by a 4 min evaporation. CH_3I (1.5 mmol) in CH_3CN (1 ml) was finally added to the dry Kryptofix 222 / K^+[^{18}F]F^- complex, and the reaction mixture was heated under reflux for 1 min. The [^{18}F]CH_3F formed was purified by gas chromatography and then mixed with approximately 1 µmol of carrier fluorine gas in Neon (0.5% F_2 / Ne) in a quartz discharge chamber. The F-19 / F-18 isotopic exchange reaction was carried out by applying a high-voltage electrical discharge (30.7 ± 1.0 kV, 10 s) through the gas mixture.

[^{18}F]Selectfluor bis(triflate) synthesis

The discharge-produced [^{18}F]F_2 was bubbled through a solution of 1-chloromethyl-4-aza-1-azoniabicyclo[2.2.2]octane triflate (1.0 ± 0.2 mg (3.2 ± 0.8 µmol)) and LiOTf (0.8 ± 0.1 mg (5.1 ± 0.6 µmol)) in acetone-d_6 (750 µl). The resulting [^{18}F]Selectfluor bis(triflate) was used in subsequent electrophilic

fluorine-18-labeling without purification and could be stored “as is” as a stock [18F]reagent solution for multiple labeling experiments.

Production of [18F]DPA-714

Aqueous [18F]fluoride from the cyclotron-target was added to a reaction vessel containing 12.8 ± 0.1 mg (34 ± 0.3 µmol) of Kryptofix 222 in 100 µl of CH_3CN and 80 µl of a 0.25 M aq. stock solution of K_2CO_3 (20 µmol). CH_3CN (0.5 ml) was then added, and the solvents were removed by azeotropic distillation, carried out for 8 min at a temperature of 100 °C under a helium flow. Two further additions of CH_3CN (0.5 ml each) were made, each followed by a 5 min evaporation. On cooling, *N,N*-diethyl-2-(2-(4-(2-tosylethoxy)phenyl)-5,7-dimethyl-pyrazolo[1,5-α]pyrimidin-3-yl)acetamide (DPA-714 labeling precursor, Fig. 1b, compound **3**, 5.6 ± 0.3 mg (10.4 ± 0.6 µmol)) in 1 ml of CH_3CN was added to the dry Kryptofix 222 / K^+[18F]F^- complex and the reaction mixture was heated under reflux for 10 min. Half of the solvent was then evaporated. The reaction mixture was finally diluted with 0.1 M aq. $CH_3CO_2NH_4$ (1.5 ml) and purified by HPLC using the same system as described for [18F]F-DPA. [18F]DPA-714 showed a retention time of about 20–22 min. Formulation was also carried out according to the same procedure as for [18F]F-DPA. HPLC analysis of [18F]DPA-714 batches was performed using the same column and eluent system as previously described for [18F]F-DPA, the retention time for [18F]DPA-714 in these conditions was 4.3 min.
